# Tcf19 is a novel islet factor necessary for proliferation and survival in the INS-1 β-cell line

**DOI:** 10.1152/ajpendo.00147.2013

**Published:** 2013-07-16

**Authors:** Kimberly A. Krautkramer, Amelia K. Linnemann, Danielle A. Fontaine, Amy L. Whillock, Ted W. Harris, Gregory J. Schleis, Nathan A. Truchan, Leilani Marty-Santos, Jeremy A. Lavine, Ondine Cleaver, Michelle E. Kimple, Dawn Belt Davis

**Affiliations:** ^1^Department of Medicine, Division of Endocrinology, Diabetes, and Metabolism, University of Wisconsin, Madison, Wisconsin;; ^2^School of Medicine and Public Health, University of Wisconsin, Madison, Wisconsin;; ^3^Department of Molecular Biology, University of Texas-Southwestern, Dallas, Texas; and; ^4^William S. Middleton Memorial Veterans Hospital, Madison, Wisconsin

**Keywords:** diabetes, β-cell, islet, proliferation, apoptosis, transcription factor 19, transcription, endoplasmic reticulum stress

## Abstract

Recently, a novel type 1 diabetes association locus was identified at human chromosome 6p31.3, and transcription factor 19 (*TCF19*) is a likely causal gene. Little is known about Tcf19, and we now show that it plays a role in both proliferation and apoptosis in insulinoma cells. Tcf19 is expressed in mouse and human islets, with increasing mRNA expression in nondiabetic obesity. The expression of *Tcf19* is correlated with β-cell mass expansion, suggesting that it may be a transcriptional regulator of β-cell mass. Increasing proliferation and decreasing apoptotic cell death are two strategies to increase pancreatic β-cell mass and prevent or delay diabetes. siRNA-mediated knockdown of Tcf19 in the INS-1 insulinoma cell line, a β-cell model, results in a decrease in proliferation and an increase in apoptosis. There was a significant reduction in the expression of numerous cell cycle genes from the late G1 phase through the M phase, and cells were arrested at the G1/S checkpoint. We also observed increased apoptosis and susceptibility to endoplasmic reticulum (ER) stress after Tcf19 knockdown. There was a reduction in expression of genes important for the maintenance of ER homeostasis (*Bip*, *p58*^*IPK*^, *Edem1*, and *calreticulin*) and an increase in proapoptotic genes (*Bim*, *Bid*, *Nix*, *Gadd34*, and *Pdia2*). Therefore, Tcf19 is necessary for both proliferation and survival and is a novel regulator of these pathways.

both type 1 diabetes mellitus (T1DM) and type 2 diabetes mellitus (T2DM) are diseases of insufficient functional β-cell mass. T1DM results from autoimmune destruction of insulin-producing pancreatic β-cells (2). In addition, apoptotic β-cell death occurs in T1DM via increased endoplasmic reticulum (ER) stress and other pathways mediated by cytokines (2, 12). There appears to be a significant period of time before the clinical onset of T1DM, during which β-cell mass is slowly declining and islet inflammation is present, resulting in a 70–80% loss of β-cell mass at the time of diagnosis (23). Additionally, there is evidence of high rates of compensatory β-cell proliferation in recent-onset T1DM (51). Therefore, regulators of β-cell proliferation and survival may play an important role in the early pathogenesis of T1DM. T2DM occurs when there is an increased demand for insulin production due to peripheral insulin resistance and insufficient functional β-cell capacity to meet the demand. Autopsy studies have shown that individuals with T2DM have reduced β-cell mass due to decreased proliferation and increased apoptosis (5, 19). There are currently no therapies for T1DM or T2DM that specifically target β-cell mass. Further understanding of the molecular mechanisms of β-cell mass regulation will be crucial in reaching this goal.

β-Cell mass increases due to proliferation, hypertrophy, and neogenesis, whereas it decreases due to apoptosis and atrophy (1). Several molecular regulators of β-cell proliferation have been identified, including growth factors, transcription factors, hormones, kinases, and cell cycle regulators (1). In addition, β-cell loss through apoptosis can occur via several pathways, including ER stress, cytokines, and oxidative stress from glucose and lipid-mediated toxicity (21). Inhibition of β-cell apoptosis has been demonstrated with several factors, including glucagon-like peptide-1 and cholecystokinin (21, 27). A smaller number of factors that can impact both β-cell proliferation and apoptosis have been identified (20). A therapeutic intervention with the capacity to impact both proliferation and survival would result in an additive benefit to β-cell mass.

We initially identified transcription factor 19 (*Tcf19*) in a microarray of islet gene expression in mouse models of obesity and diabetes. Within this model, obesity is induced in both the C57BL/6J (B6) and the BTBR strains via introduction of the *ob* mutation (Lep^*ob*^) into the leptin gene. Despite the presence of obesity and insulin resistance, the B6 *ob*/*ob* mouse is resistant to diabetes. However, the BTBR *ob/ob* mouse develops significant hyperglycemia by 10 wk of age and has reduced islet mass and proliferation compared with the B6 *ob/ob* mouse (22). *Tcf19* was identified within a group of coordinately regulated genes enriched for cell cycle gene ontology. The expression pattern of these genes correlated with islet proliferation in these mouse models (22).

Tcf19, then referred to as SC1, was first described in 1991 as a putative transactivating factor, with expression beginning at the late G1/S boundary in dividing cells (25). Despite its initial identification more than two decades ago, it remains essentially uncharacterized. Recently, *TCF19* was genetically associated with T1DM, but the role of *TCF19* in T1DM pathogenesis remained unclear (8). Analysis of the primary sequence of Tcf19 reveals a forkhead association (FHA) domain, which may serve as a nuclear signaling domain or as a phosphoprotein binding domain (11, 25, 30). Notably, proteins containing an FHA domain include several well-known cell cycle proteins such as Ki-67 and Chk2 (11, 30). Tcf19 also contains a proline-rich region, a common characteristic of transactivating factors. Additionally, human Tcf19 contains a PHD or RING finger region at its carboxyl terminus, which may allow it to interact with chromatin via methylated histone H3 (25, 42). These characteristics support a role for Tcf19 as a cell cycle and transcriptional regulator. Combining these traits with our observation that *Tcf19* correlates with islet proliferation in obesity and with cell cycle gene expression in mouse islets (22), we hypothesized that Tcf19 is a transcriptional regulator of β-cell mass.

To develop this hypothesis, we first needed to learn more about the expression and the function of *Tcf19*. We found that Tcf19 is expressed in mouse and human islet and upregulated with obesity. We next used knockdown experiments to demonstrate that Tcf19 is necessary for growth and survival in the INS-1 cell line. Finally, we examined changes in gene expression after Tcf19 knockdown and identified genes in cell cycle regulation and maintenance of ER homeostasis as potential transcriptional targets of Tcf19. Overall, we identify Tcf19 as a novel transcription factor important in growth and survival.

## MATERIALS AND METHODS

### 

#### Cell culture.

INS-1 cells were cultured in RPMI 1640 supplemented with 1% antibiotic-antimycotic (Gibco, 15240–062), 1% l-glutamine, 1% sodium pyruvate, and 10% fetal bovine serum. 2-Mercaptoethanol was added to a final concentration of 0.352% to supplemented media before each use. Cells were cultured in RPMI lacking serum and containing 2 mM glucose for 24 h prior to harvest for serum starvation experiments.

#### Animals.

cDNA from 10-wk-old male lean and obese (leptin^*ob*^ mutation homozygotes; *ob*/*ob*) C57BL/6 (B6), and BTBR mice was generously provided by Alan Attie. We further isolated additional tissue from 10-wk-old lean and *ob*/*ob* B6 mice. Mouse protocols were approved by the University of Wisconsin Animal Care and Use Committee to meet acceptable standards of humane animal care.

#### Human islet BMI panel.

Human islets were obtained from nondiabetic organ donors through the Integrated Islet Distribution Program, including the Centers at Scharp/Lacy, Emory University, University of Illinois, Massachusetts General, University of Southern California, University of Miami, University of Pennsylvania, University of Minnesota, and University of Wisconsin. Human islets were processed for RNA within 24 h after arrival of the shipment. An exemption was granted for human islet work by the Institutional Review Board at the University of Wisconsin. Human insulinoma tissue was generously provided by Herbert Chen and was obtained under approval from the Institutional Review Board at the University of Wisconsin.

#### Adenoviral experiments.

Human and mouse islets were incubated with adenovirus containing FoxM1 or β-galactosidase genes, as described previously (10).

#### Western blotting.

INS-1 cells were harvested 3 days after transfection and washed in ice-cold PBS. Cells were lysed in 20 mM Tris·HCl, 10 mM EDTA, and 1% NP-40 containing protease inhibitors. Whole cell lysate was mixed with NuPAGE sample loading buffer (Invitrogen) containing DTT and then separated on a 4–10% SDS-PAGE gel and transferred to a polyvinylidene difluoride membrane. The membrane was blocked in 5% milk in Tris-buffered saline with 0.1% Tween 20. Blots were developed with ECL Prime (Amersham), imaged with a charge-coupled device camera, and then quantitated by densitometry with Image J 1.44o (http://imagej.nih.gov/ij) (15). The percent reduction in expression was determined for each transfection and averaged. Results were compared by paired *t*-test, and significance was determined by a *P* value of <0.05. Primary antibodies and dilutions were as follows: Tcf19, SC-69026 at 1:500 (Santa Cruz Biotechnology), cyclin D1, MS-210 at 1:200 (Thermo Scientific), β-tubulin, SC-9104 at 1:1,500 (Santa Cruz Biotechnology).

#### In situ hybridization.

A 1,600-bp digoxigenin-RNA *Tcf19* probe was generated from clone BC004617 (Open Biosystems) following linearization with *Sal*1 and polymerization using T7. CD1 embryos were collected from pregnant females [embryonic day E10.5 through E15.5] by dissection in ice-cold PBS buffer and fixed overnight in 4% paraformaldehyde. Adult tissue was collected and fixed in a similar manner. Whole mount in situ hybridization was carried out with modifications from D. Wilkinson's method, as described previously in (46, 50). In situ hybridization on tissue sections was performed on 10-μm sections from paraffin-embedded tissue. Sections were deparaffinized and treated for 10 min with 15 μg/ml proteinase K. Sections were refixed in 4% paraformaldehyde for 5 min and then rinsed three times in PBS. Prehybridization was carried out in a chamber humidified with 50% formamide-5× saline-sodium citrate (SSC), and a bolus containing hybridization buffer was left on the slides for 1 h at room temperature. Probe hybridization (probe at 1 μg/ml) was done with a 100-μl probe/slide (covered with glass coverslips) at 65°C overnight. Slides were placed posthybridization in 5× SSC at 65°C to allow coverslips to separate. Slides were rinsed twice in 0.2× SSC at 72°C for 30 min and once in 0.2× SSC at room temperature for 5 min and then in MBST buffer at room temperature (100 mM maleic acid, 150 mM NaCl, pH 7.5, 0.1% Tween-20). Slides were incubated in blocking solution [2% blocking reagent (Roche) in MBST] for 1 h at room temperature. Anti-Dig alkaline phosphatase-conjugated antibody was applied on slides in a chamber humidified with MBST [250 μl of 1/4,000 anti-Dig antibody (Roche)], covered with parafilm, and incubated overnight at 4°C. Slides were washed for 3 × 30 min in MBST after antibody incubation and treated in NTMT (100 mM NaCl, 100 mM Tris, pH 9.5, 50 mM MgCl2, and 0.1% Tween-20) for 3 × 5 min. Color reaction was carried out using BM purple (Roche).

#### Transfection with siRNA.

INS-1 cells were transfected with either Tcf19 siRNA (ID no. s98717; Ambion) or a scrambled control siRNA (Ambion). Transfections were performed with Lipofectamine 2000 (Invitrogen). INS-1 cells were trypsinized and resuspended in transfection medium (RPMI 1640 supplemented with 1% l-glutamine, 1% sodium pyruvate, and 10% fetal bovine serum). Cells in transfection medium were then added to an siRNA (final concentration of 47 nM)-lipofectamine mixture and plated. Transfection medium was removed 12–18 h posttransfection and replaced with supplemented RPMI, as described above. Medium was changed every 48 h thereafter. Each transfection was also performed in technical triplicate (3 separate wells/treatment group plated). Transiently transfected INS-1 cells were harvested 3–7 days posttransfection. Reported *n* for each experiment is the number of separate transfections performed. Technical replicate results were averaged to give a single value for each transfection replicate.

#### Quantitative real-time PCR.

RNA was isolated from INS-1 cell pellets harvested 3 days posttransfection or from mouse or human tissues using the RNeasy Kit (Qiagen). Concentration and purity of RNA was determined using a NanoDrop ND-1000 Spectrophotometer, and cDNA was synthesized using the SuperScript VILO cDNA Synthesis Kit (Invitrogen). Quantitative real-time PCR reactions were carried out using Power SYBR green PCR Master Mix (Applied Biosystems) and the StepOnePlus Real Time PCR System (Applied Biosystems). Primer sequences are available upon request.

Cycle time (C*t*) values were normalized to β-actin to yield a ΔC*t*. Fold changes were then calculated between experimental and control samples: fold change = 2^(ΔC*t*_experimental_ − ΔC*t*_control_). Percent change in mRNA expression was calculated as %change = 100 × (1 − fold change). Results were analyzed by paired *t*-test of the ΔC*t* values, and significance was determined by *P* < 0.05.

#### Glucose-stimulated insulin secretion.

Glucose-stimulated insulin secretion was measured in transiently transfected INS-1 using an insulin ELISA. Three days posttransfection, cells were preincubated in KREBS buffer solution for 2 h. After the incubation, cells were then incubated at either 3 or 16.7 mM glucose for an additional 2 h. Stimulation medium was harvested and kept in separate tubes, whereas the cells were lysed with acid ethanol for cellular insulin content measurement. All samples were stored at −20°C until they were processed. Insulin was measured using ELISA, as described previously (27). Results were compared by unpaired *t*-test, and significance was determined by *P* < 0.05. Antibodies and dilutions are as follows: monoclonal capture antibody (insulin antibody clone D6C4; Research Diagnostics) at 1:3,000 and biotinylated anti-insulin-detecting antibody (insulin antibody clone D3E7; Research Diagnostics) at 1:2,000.

#### Viability and proliferation.

Transiently transfected INS-1 cells were harvested on *days 3–7* posttransfection, and cell viability was determined using the Vi-Cell XR Viability Analyzer (Beckman Coulter). Technical triplicates were plated in separate wells for each transfection, and a total of five separate transfections were done. Comparisons were made by unpaired *t*-test, including all technical and biological replicates; statistical significance was determined by *P* < 0.05.

To measure cell proliferation, transiently transfected INS-1 cells were incubated with ^3^[H]thymidine (NET027001MC; Perkin Elmer) at a final concentration of 1 μCi/ml in supplemented RPMI for 4 h. Again, technical triplicates were plated in separate wells for each transfection; three to four transfections were performed at each time point. Cells were then trypsinized and washed three times with ice-cold PBS. DNA and protein were precipitated by the addition of ice-cold 10% trichloroacetic acid and incubated for 30 min on ice. The precipitate was then pelleted at 18,000 *g* for 10 min at 4°C. Pelleted precipitate was solubilized in 0.3 N NaOH and vortexed for 15 min. Radioactivity was measured using a liquid scintillation counter, and a fraction of the solubilized product was kept to measure total protein by the Bradford assay (3). Sample counts were individually normalized to protein, and an average for each transfection was determined. Results were analyzed by paired *t*-test, and statistical significance was determined by *P* < 0.05.

#### Flow cytometry.

Apoptosis was measured in transiently transfected INS-1 cells using Annexin V and propidium iodide staining in live cells. Three days posttransfection, cells were treated with either 1 μM thapsigargin (TG) or vehicle (0.01% DMSO) for 20–24 h. Cells were then harvested and washed once in ice-cold PBS. Next, cells were washed in 1 ml of annexin-binding buffer (10 mM HEPES, 140 mM NaCl, and 2.5 mM CaCl_2_, pH 7.4) and resuspended in annexin-binding buffer to a density of 1 × 10^6^ cells/ml. Cell suspension was then incubated in the dark with Alexa Fluor 488-annexin V (1:20, A13201; Molecular Probes) and propidium iodide (50 μg/ml) for 15 min at room temperature. Samples were kept on ice and immediately read on a BD FACSCalibur flow cytometer. Data was analyzed with FlowJo version 9.2, and results were compared by paired *t*-test. Statistical significance was determined by *P* < 0.05.

Cell cycle progression was measured in transiently transfected INS-1 cells, using propidium iodide staining of fixed cells. Cells were harvested 3 days posttransfection, and 1 × 10^6^ cells were resuspended in PBS. Cells were then fixed for 30 min at −20°C with chilled 70% EtOH and added dropwise to cell pellets with gentle vortexing. Cells were washed once in PBS and resuspended in propidium iodide staining solution (1 mg/ml RNase A, 33 μg/ml propidium iodide, and 0.1% NP-40 in PBS at pH 7.4 without Ca^2+^, without Mg^2+^, with 0.1% BSA, and with 1 mM EDTA). Cells were then incubated in the dark at room temperature for 30 min before analysis on a BD FACSCalibur flow cytometer. Data were analyzed with ModFit software, and results were compared by paired *t*-test. Statistical significance was determined by *P* < 0.05.

## RESULTS

### 

#### Tcf19 is expressed in pancreatic islets.

**Fig. 1.**
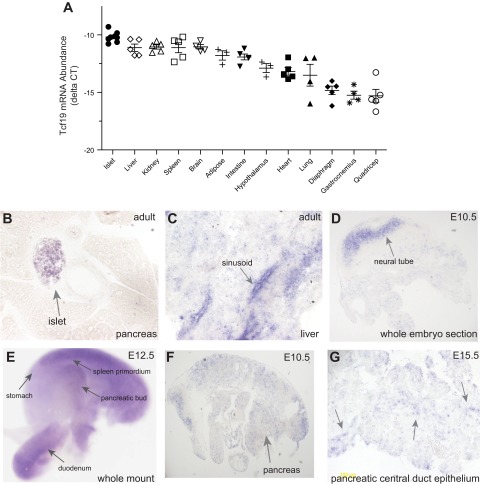
*Tcf19* expression is most highly expressed in islet and is present at low levels in mouse embryonic pancreas. *A*: quantitative RT-PCR analysis of *Tcf19* expression across 13 tissues in C57BL/6 (B6) mouse at 8–10 wk old. Cycle time (C*t*) values were normalized to β-actin to yield ΔC*t* values; *n* ≥ 3. *B–G*: in situ hybridization with a full-length mouse Tcf19 probe. *B*: adult pancreas at ×20 magnification shows that Tcf19 expression is enriched in the islet but not in exocrine tissue. There is also some staining in cells of the nearby duct. *C*: adult liver expresses Tcf19 in sinusoid regions. *D*: whole embryo section at embryonic day E10.5 demonstrates predominant expression in the neural tube. *E*: whole mount in situ of E12.5 embryo demonstrates mesenchymal expression. *F*: whole embryo from E10.5 is shown at ×2.5 magnification after longer exposure, with an arrow indicating region of the pancreas. *G*: higher-magnification view of pancreatic duct epithelium (ribbon-like structure indicated by arrows) in E15.5 mouse pancreas showing very low-level staining.

Little is known about Tcf19 tissue distribution in adult organisms. Given our hypothesis that Tcf19 plays a role in β-cell mass regulation, we focused further expression studies on the pancreas and pancreatic islet. Microarray studies indicate high *TCF19* expression in human pancreas (52). To determine the tissue distribution of *Tcf19*, we collected 13 tissues from lean B6 mice. Quantitative RT-PCR analysis revealed that *Tcf19* expression is high in islets at levels comparable with the expression in several other tissues such as liver, kidney, spleen, and brain (Fig. 1*A*).

We next examined adult mouse pancreas with in situ hybridization and found that *Tcf19* is expressed predominantly in the islets rather than in acinar or ductal tissue (Fig. 1*B*). We observed a few ductal cells expressing *Tcf19* (Fig. 1*B*). In accord with the PCR data, *Tcf19* was also expressed at relatively high levels in the adult liver sinusoids (Fig. 1*C*).

During development, some groups have identified varying levels of *Tcf19* expression in regions of the brain from days E10.5 to 16.5, whereas others found globally low expression in whole mount or sectioned mouse embryo (16, 43, 48). We sought to clarify expression of *Tcf19* in the developing mouse embryo, with a specific focus on pancreatic islet expression. We generated a full-length probe of mouse *Tcf19* and examined mouse embryos from E10.5 to E15.5 with in situ hybridization. We saw robust staining in the embryonic neural tube at E10.5 (Fig. 1*D*). Otherwise there was generally low-level expression in the mouse embryonic tissues, including the pancreas. Whole mount E12.5 embryo also showed neural tube and mesenchymal expression (Fig. 1*E*). Upon closer examination and with longer exposure times, we were able to detect low-level *Tcf19* expression in disperse nuclei throughout the gut on days E10.5, E12.5, and E15.5 (Fig. 1*F* and data not shown). The pancreatic expression was very faint, but when present it was detected along the central duct epithelium at E15.5, which is the location of the delaminating islet progenitors (arrows in Fig. 1*G*). Our work confirms that *Tcf19* is highly expressed during early development in the neural tube but overall has globally low expression in the mouse embryo. However, in focusing on the developing pancreas we found that *Tcf19* is expressed at very low levels but can be detected in the developing islet cells.

#### Tcf19 is upregulated in obesity and in islet proliferation.

**Fig. 2.**
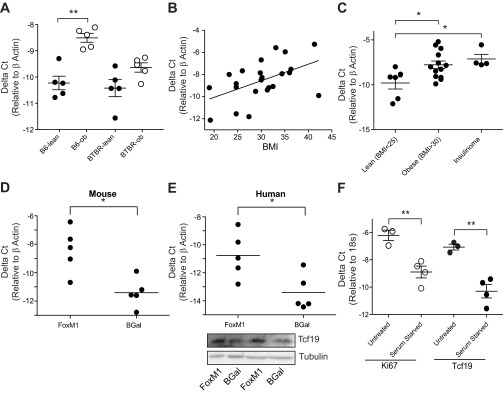
*Tcf19* islet expression is preferentially upregulated in nondiabetic obesity and in proliferating islets. *A*: quantitative RT-PCR analysis of *Tcf19* expression in islet from 10-wk-old lean (●) and obese (*ob*/*ob*; ○) B6 and BTBR animals. C*t* values were normalized to β-actin to yield ΔC*t* values. Comparisons were made by unpaired *t*-test; *n* = 5. *B*: quantitative RT-PCR analysis of *TCF19* expression in human islets as a function of BMI; *r*^2^ = 0.2572 and *P* = 0.0135. *C*: quantitative RT-PCR analysis of TCF19 expression in lean (BMI < 25) or obese (BMI ≥ 30) human islets and human insulinoma tissue. Comparisons were made by unpaired *t*-test. *D*: quantitative RT-PCR analysis of *Tcf19*/*TCF19* expression in mouse and human islets after treatment with adenovirus to induce overexpression of FoxM1 or control β-galactosidase adenovirus. Comparisons were made by paired *t*-test; *n* = 5. *E*: Western blot analysis of TCF19 in human islets treated with FoxM1 or control β-galactosidase adenovirus. Two representative biological replicates are shown. Tubulin is shown as loading control. *F*: INS-1 were serum starved for 24 h, and then *Ki67* and *Tcf19* gene expression was analyzed by quantitative RT-PCR. C*t* values were normalized to 18s to yield ΔC*t* values. Comparisons were made by unpaired *t*-test; *n* ≥ 3. **P < 0.01; *P < 0.05.

To confirm the regulation of *Tcf19* expression by nondiabetic obesity initially identified in a microarray study (22), we used quantitative RT-PCR to measure *Tcf19* expression in response to obesity at 10 wk of age in both the B6 and BTBR strains of mice. We observed a 3.3-fold increase in *Tcf19* mRNA expression (*n* = 5, *P* = 0.0005) with obesity in the nondiabetic B6 strain, but this obesity-driven upregulation was not present in the diabetic BTBR strain (Fig. 2*A*). Analysis of *Tcf19* mRNA expression in liver tissue from these mice revealed no difference in expression across any of the four mouse strains (data not shown). Review of previous microarray analyses also showed no changes in *Tcf19* expression in liver, skeletal muscle, or hypothalamus across strains (22). *Tcf19* was upregulated in adipose tissue from 10-wk-old *ob*/*ob* animals in both the B6 and the BTBR strains (22). Therefore, there appears to be tissue-specific regulation of *Tcf19* expression. Next, we analyzed *TCF19* expression in human islet samples from nondiabetic donors and identified a positive correlation of *TCF19* expression with BMI (BMI range 19.0–42.2, *r*^2^ = 0.257, *P* = 0.0135, *n* = 23; Fig. 2*B*). Dividing donors into lean (BMI < 25) and obese (BMI ≥ 30) categories, we observed a 4.1-fold increase in *TCF19* expression (*P* = 0.0167), similar to the upregulation seen in the B6 mouse with obesity (Fig. 2*C*). We saw no difference in age, sex, race, cold ischemia time, or time to shipment between the obese and nonobese donors. There was also no correlation of *TCF19* expression with age of donors (*r*^2^ = 0.0117 and *P* = 0.6230, age range 16–65 yr; *n* = 23). These data establish a positive correlation between mouse and human islet *Tcf19* expression and nondiabetic obesity.

We next wanted to look directly at expression of Tcf19 in proliferating islet tissue. *TCF19* is expressed in human insulinoma at levels similar to normal islets from obese donors (Fig. 2*C*), suggesting that it is not highly upregulated in β-cell tumors. We have shown previously that adenoviral overexpression of FoxM1 is able to drive β-cell proliferation in mouse and human islet through upregulation of numerous cell cycle genes (10). In mouse islets overexpressing FoxM1, there is a 12.8-fold increase in *Tcf19* expression, and in human islets that overexpress FoxM1 there is a 10.1-fold increase in *TCF19* expression (Fig. 2, *D* and *E*). This upregulation of gene expression is corroborated by an increase in Tcf19 protein expression with FoxM1 overexpression in human islet (Fig. 2*E*).

To further establish the direct link between Tcf19 and cell division and replicate the growth regulation of *Tcf19* expression seen in 3T3 cells (25), we serum starved INS-1 cells for 24 h. Serum-starved cells had reduced proliferation, as indicated by an 84% reduction in Ki-67 gene expression (*P* = 0.006). Growth arrest from serum starvation resulted in an 89% reduction in Tcf19 gene expression (*P* = 0.003; Fig. 2*F*).

#### Tcf19 is necessary for proliferation in INS-1 cells.

**Fig. 3.**
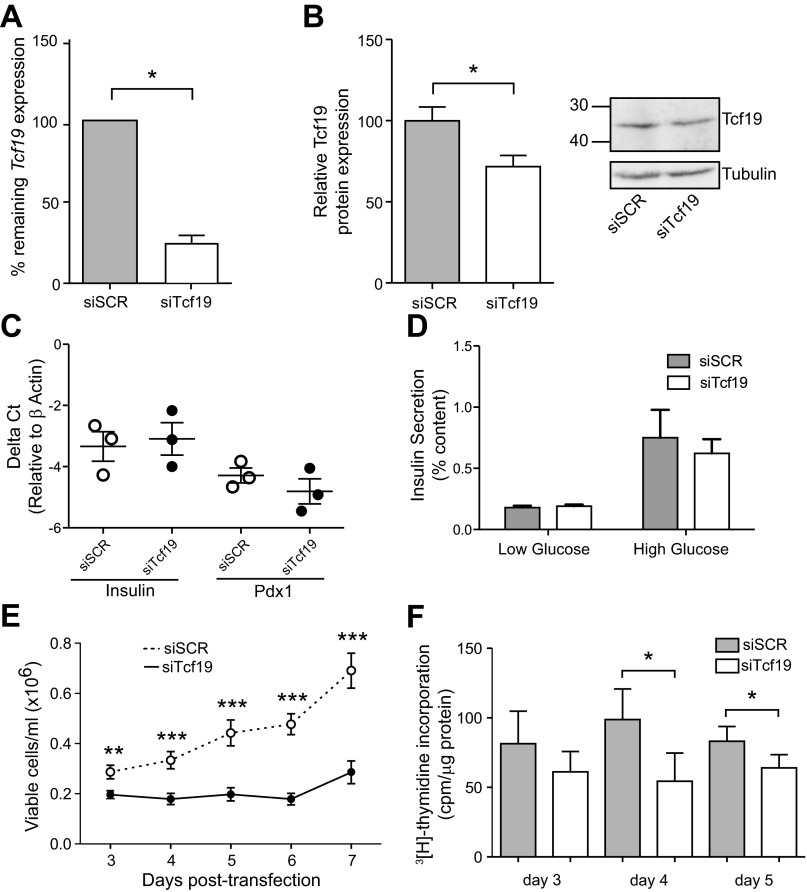
*Tcf19* knockdown results in reduced cell growth and proliferation. *A*: transient transfection of INS-1 cells results in a 76% mean reduction in *Tcf19* mRNA expression on *day 3* posttransfection in cells transfected with siTcf19 vs. siSCR (scrambled control), shown by quantitative RT-PCR analysis. C*t* values were normalized to β-actin to yield ΔC*t* values. Comparison was made by paired *t*-test. *P* = 0.02; *n* = 3. *B*: Western blot analysis reveals 44% reduction in Tcf19 protein, quantified by densitometry. Comparison was made by paired *t*-test. *P* = 0.04; *n* = 3, representative blot shown. *C*: no changes in expression of insulin (*Ins1*) and *Pdx1* in INS-1 cells after Tcf19 knockdown, measured by quantitative RT-PCR relative to β-actin. *D*: no changes in basal (low = 3 mM glucose) or glucose-stimulated insulin secretion (high = 16.7 mM) in INS-1 cells after Tcf19 knockdown. Data are means ± SE; *n* = 4. *E*: viable cells/ml of culture media were measured on *days 3–7* posttransfection with siTcf19 or siSCR siRNA. Comparisons were made by unpaired *t*-test; *n* = 5. *F*: Tcf19 knockdown reduces proliferation. [^3^H]thymidine incorporation analysis of INS-1 cells transfected with siTcf19 or siSCR. Comparisons were made by paired *t*-test; *n* = 4 (*day 3*) and *n* = 3 (*days 4* and *5*). *P < 0.05; **P < 0.01; ***P < 0.001.

Because numerous rodent models demonstrate compensatory increases in islet mass in response to metabolic demand (1), we hypothesized that Tcf19 plays an important role in regulation of obesity-driven β-cell proliferation. However, since β-cells have very low proliferative rates, it is difficult to study β-cell proliferation in isolated islets. To examine the direct role of Tcf19 in cell cycle progression, we used siRNA-mediated knockdown of *Tcf19* in INS-1 cells, a rat insulinoma cell line. INS-1 cells express Tcf19 protein at a level that is similar to the mouse β-cell line MIN6, and both cell lines have moderately increased Tcf19 protein relative to mouse islets (data not shown). By *day 3* posttransfection in the INS-1, *Tcf19* mRNA expression was reduced by 76% in transiently transfected cells (Fig. 3*A*), which translated to a 44% decrease in Tcf19 protein expression (Fig. 3*B*). This reduction in Tcf19 did not appear to induce dedifferentiation or dysfunction of the cells, as demonstrated by no change in insulin or Pdx1 gene expression (Fig. 3*C*). Glucose-stimulated insulin secretion is also unaffected by Tcf19 knockdown (Fig. 3*D*). However, following siRNA-mediated knockdown of Tcf19 in INS-1 cells, we observed a significant reduction in the number of viable cells on *days 3–7* posttransfection. (Fig. 3*E*).

The two major processes that impact on cell number are proliferation and apoptosis. To determine the role of Tcf19 in INS-1 cell proliferation, we used a ^3^[H]thymidine incorporation assay in cells that had been transfected with the Tcf19 or control siRNA. With reduced Tcf19 expression, we observed a 45 and 23% reduction in proliferation on *days 4* and *5* posttransfection, respectively (Fig. 3*F*). These data demonstrate that Tcf19 is necessary for normal proliferation of INS-1 cells.

#### Tcf19 knockdown affects cell cycle gene expression and is necessary for proper cell cycle progression.

**Fig. 4.**
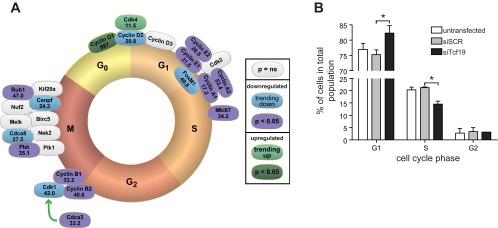
Tcf19 knockdown results in reduced expression of many cell cycle genes and disrupts normal cell cycle progression. *A*: measurements were obtained by quantitative RT-PCR. C*t* values were normalized to β-actin to yield ΔC*t* values. Comparisons were made by paired *t*-test; *n* = 3. The color of each gene oval represents the *P* value, as indicated in the legend. Green ovals represent upregulated genes, blue/purple ovals represent downregulated genes, and gray ovals represent unchanged genes. Trending indicates a *P* value between 0.1 and 0.05. The number inside each oval is the %change in expression in INS-1 cells treated with siTcf19 vs. siSCR control. *B*: cell cycle phase measured by propidium iodide staining of DNA and flow cytometry. siTcf19 is after Tcf19 knockdown and siSCR is after scrambled control. *P < 0.05, *n* = 3. NS, not significant.

To further explore the impact of Tcf19 on INS-1 cell proliferation, we examined the effects of Tcf19 knockdown on the expression of genes important throughout all phases of the cell cycle. Quantitative RT-PCR analyses revealed significant changes in the expression of numerous cell cycle genes (Fig. 4*A*). At the G1/S transition, we observed a 26–32% decrease in the expression of E-type cyclins and a 17–53% reduction in expression of A-type cyclins, which also function during the G2/M transition. We also observed decreased expression of B-type cyclins (33–41%) and *Cdca3* (33%) during the G2/M transition (Fig. 4*A*). In the M phase, expression of two key mitotic kinases, *Bub1* and *Pbk*, was reduced by 47 and 65%, respectively. Notably, we also observed a 34% decrease in the expression of the proliferation marker *Mki67*. Analysis of the cell cycle inhibitors *p14/16*, *p15*, *p18*, *p19*, *p21*, *p27*, and *p57* did not reveal any significant difference in gene expression after Tcf19 knockdown (data not shown). Collectively, these data show that Tcf19 is an important regulator of the expression of select cell cycle genes.

In contrast to other cell cycle gene expression changes, we detected an 887% (9.87-fold) increase in *CcnD1* expression in response to Tcf19 knockdown (Fig. 4*A*). However, there was no statistically significant change in the mRNA levels of its binding partner *Cdk4* or in the expression of *cyclins D2* and *D3*. Furthermore, Western blot revealed no change in cyclin D1 protein expression in response to Tcf19 knockdown (quantified with densitometry compared with β-tubulin loading control, relative density siTcf19, 1.02 ± 0.04 vs. siScr, 1.08 ± 0.21, *P* = 0.65, *n* = 3; data not shown).

Given these gene expression changes, we hypothesized that following Tcf19 knockdown, cells would be arrested at late G1 or at the G1/S interphase. Notably, *Tcf19* first begins to be expressed during the late G1 phase of cell division in synchronized cells (25). We used flow cytometry to examine the DNA content and identify cell cycle phase in INS-1 cells after Tcf19 knockdown. We observed a 7.0% increase in the fraction of cells in the G1 phase and a concomitant 6.7% decrease in the fraction of S phase cells with Tcf19 knockdown (Fig. 4*B*). This significant decrease in the portion of cells that are able to progress through the G1/S transition demonstrates that Tcf19 is necessary for cell cycle progression beyond the G1/S checkpoint.

#### Tcf19 is necessary for cell survival and regulates expression of ER stress genes.

**Fig. 5.**
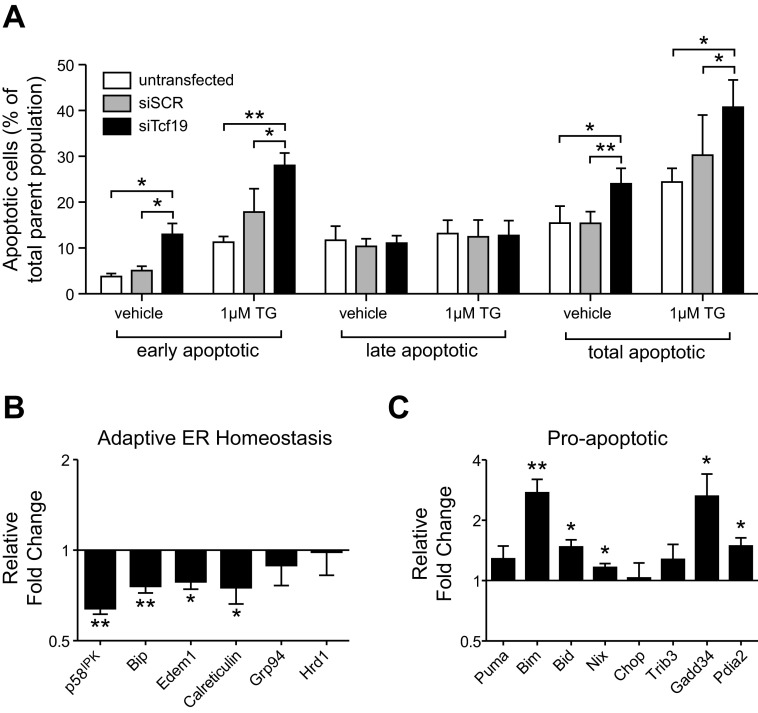
Tcf19 knockdown reduces β-cell survival and changes adaptive endoplasmic reticulum (ER) stress and proapoptotic gene expression. *A*: total apoptotic cells and %cells in early and late apoptosis, as measured by annexin V and propidium iodide staining and flow cytometry; *n* = 4. *B* and *C*: quantitative RT-PCR analysis of genes involved in adaption to ER stress and maintenance of ER homeostasis (*B*) and analysis of pro-apoptotic genes (*C*). C*t* values were normalized to β-actin to yield ΔC*t* values. Comparison was made by paired *t*-test; *n* = 5. *P ≤ 0.05; **P < 0.01. TG, thapsigargin.

The modest changes in cell proliferation observed do not entirely explain the significantly lower number of viable cells seen after Tcf19 knockdown. Therefore, we also measured apoptosis using staining for annexin V and propidium iodide. We found that Tcf19 knockdown caused a 2.57-fold increase in the fraction of early apoptotic cells (Fig. 5*A*). Given the high susceptibility of β-cells to ER stress, we further examined apoptotic rates with induction of ER stress using TG. With Tcf19 knockdown, TG treatment led to increased apoptosis (28.03% of cells in early apoptosis with TG + siTCF19 compared with 17.85% in early apoptosis with TG + siSCR; Fig. 5*A*). These data suggest that Tcf19 protects against apoptosis and reveal a role for Tcf19 as a potential regulator during early apoptotic events, particularly in the setting of ER stress.

To assess downstream transcriptional effects mediated by Tcf19, we next examined the expression of genes that are transcriptionally regulated in the ER stress response after Tcf19 knockdown. We observed a significant decrease in expression of several genes involved in the adaptive unfolded protein response (UPR), including a 27 and 36% decrease in expression of the ER chaperone *Bip* (*HspA5*) and its cochaperone *p58*^*IPK*^ *(Dnajc3)*, respectively (Fig. 5*B*). We also measured a 22% decrease in the expression of *Edem1*, which plays a role in targeting misfolded glycoproteins for degradation by the endoplasmic reticulum-associated degradation (ERAD) pathway, and a 27% decrease in the expression of *calreticulin (Calr)*, a cytosolic glycoprotein chaperone (9, 17, 32, 35–37) (Fig. 5*B*). A decrease in expression of this group of genes with knockdown of Tcf19 suggests that Tcf19 functions as a transcriptional activator of the adaptive UPR response to ER stress.

Conversely, we also observed a significant increase in the expression of numerous proapoptotic ER stress genes. We observed a 141 and 45% increase in the expression of *Bim* (*Bcl2l11*) and *Bid*, respectively, both of which are members of the BCL-2 family and activate Bax/Bak-mediated apoptosis. In addition, we found a 16% increase in expression of *Nix (Bnip3l)*, which has been shown to localize to the ER to stimulate Bax/Bak-independent apoptosis or to mitochondria to stimulate Bax/Bak-dependent apoptosis (Fig. 5*C*) (6, 14, 33, 41). Expression of *Gadd34* (*Ppp1r15a*), which functions as a negative regulator of the UPR by derepressing eIF2α activity and allowing translation of stress-induced gene products, increased 145% with Tcf19 knockdown (Fig. 5*C*) (4, 24, 29, 34, 53). Additionally, we saw a 46% increase in the expression of *Pdia2*, a protein disulfide isomerase that acts as a retention factor, limiting pro-insulin egress from the ER (Fig. 5*C*) (39). Upregulation of these genes in response to Tcf19 knockdown implies that the balance has been tipped from the adaptive UPR to ER stress-induced apoptosis.

## DISCUSSION

In this article we have identifed a novel gene, *Tcf19*, in the pancreatic islet and have described its importance in cell proliferation and survival. We find that *Tcf19* expression is highly correlated with proliferation in the islet. We further show that Tcf19 is necessary for growth and survival in INS-1 cells through transcriptional regulation of key cell cycle genes and genes involved in apoptotic pathways.

We propose that Tcf19 is involved in cell cycle progression and proliferation in the pancreatic β-cell. We provide evidence that *Tcf19* expression is positively correlated with islet expansion in nondiabetic obesity (Fig. 2, *A* and *B*) and that Tcf19 expression increases in response to FoxM1 overexpression, a known driver of β-cell proliferation (Fig. 2, *D* and *E*). We have previously identified a number of key cell cycle genes whose expression is also positively correlated with BMI in human islets (10). We proposed that these genes were critical in the obesity-driven expansion of β-cell mass via proliferation. In a β-cell line model, Tcf19 knockdown results in a decrease in proliferation (Fig. 3*F*) that corresponds with decreased expression of many of these same cell cycle genes, specifically including those that regulate the G1/S transition and are known to be key factors in β-cell proliferation (Fig. 4*A*) (18, 44). The result was induction of a G1/S cell cycle arrest (Fig. 4*B*). We saw what was likely a compensatory upregulation in *cyclin D1* mRNA expression with no change in cyclin D1 protein expression, which was perhaps due to global translational inhibition from ER stress (Fig. 4*A* and data not shown) (7). This pattern of cell cycle gene regulation is similar to that seen with other transcription factors that regulate β-cell proliferation via effects on A and E cyclins without having direct effects on D cyclin expression, including FoxM1 and Nkx6.1 (10, 40). Together, these data strongly suggest that not only is Tcf19 necessary for β-cell proliferation but that it may be an important transcriptional regulator of the G1/S transition via effects on A and E cyclins. It may mediate these effects through interaction with FoxM1 protein and direct transcriptional regulation of the *FoxM1* transcript and may itself be a FoxM1 target gene.

In addition to its role in proliferation, Tcf19 is also important for cell survival. We found a significant increase in INS-1 cell apoptosis both at baseline and under ER stress with Tcf19 knockdown (Fig. 5*A*). The pancreatic β-cell has a high secretory capacity, and therefore, its ER is under constant strain. During physiological levels of ER stress, the β-cell adapts with increased expression of ER chaperones to improve protein folding, global decreases in protein translation, and ERAD and export of excess unfolded protein (53). Many of these adaptive UPR genes are regulated by the transcription factors XBP1 and ATF6 (45). We examined the effects of Tcf19 knockdown on several transcriptionally regulated genes involved in the UPR primarily selected as genes that were known to change in response to ER stress signals and/or diabetes in the islet (38, 49). We found an overall decrease in several genes important in the adaptive UPR, including *Bip*, *p58*^*IPK*^, *Edem1*, and *calreticulin* (Fig. 5*B*). Taken together, a decline in these key mediators of proper protein folding and transport in the ER can lead to increased ER stress and apoptosis. At least one of these genes is of particular importance in β-cell survival. *P58*^*IPK*^ knockout mice display increased islet apoptosis and become insulin deficient, mimicking diabetes-related β-cell failure (26). *P58*^*IPK*^ is differentially expressed in a mouse model of systemic insulin resistance and diabetes, and its expression increases during saturated fatty acid-induced apoptosis in both mouse and human islet (28, 31). Tcf19 appears to be critical for adequate expression of these ER homeostasis genes and may act in concert with other transcriptional regulators, such as ATF6.

With Tcf19 knockdown we also see a global increase in the effectors of β-cell apoptosis, including *Bim*, *Bid*, *Nix*, *Gadd34*, and *Pdia2* (Fig. 5*C*). Again, several of these genes have documented importance to β-cell apoptosis specifically. Nix is a member of the BCL-2 family and has been shown to be necessary for β-cell apoptosis in the setting of Pdx1 insufficiency (14). Pdia2 overexpression in β-cells can lead to ER stress due to an “unfoldase” activity on proinsulin in the ER and is actually a retention factor for proinsulin in the ER (39, 55).

Therefore, knockdown of Tcf19 leads to a general upregulation of proapoptotic gene transcription. Combining this with its effects on adaptive UPR gene transcription, we hypothesize that Tcf19 functions as a transcriptional activator of UPR adaptive genes. In the absence of this activation, the reduced levels of ER chaperones and ERAD pathway members may tip the balance from adaptive UPR to proapoptotic ER stress and predispose the β-cell to apoptosis. The upregulation of these proapoptotic genes may be an indirect consequence of disrupted ER homeostasis or a direct inhibitory effect of Tcf19 on the transcription of these genes via effects on ATF3, ATF4, or CHOP activity.

Given its initial description as a growth-regulated gene (25), we predicted that Tcf19 would be expressed only in proliferative tissues. Surprisingly, we found that *Tcf19* was highly expressed in the adult islet (Figs. 1*A* and 2, *A*–C*)*, which has very low basal proliferative rates (5, 10, 13). The expression of *TCF19* in human insulinoma tissue was not significantly above that in normal islet tissue from obese donors, suggesting that Tcf19 is not dramatically upregulated in this form of cancer. In addition, we did not see globally high levels of expression of Tcf19 in the proliferative tissues of a developing mouse embryo. Tcf19 is expressed at low levels in the developing mouse pancreas (Fig. 1, *D–G*). Although low expression of a transcription factor during development does not necessarily exclude its importance in islet differentiation, the expression levels we observed in pancreas are much lower than those seen for key β-cell differentiation transcription factors such as Pdx1 or neurogenin 3 (46, 47). Therefore, this expression pattern suggests that islet expression of Tcf19 may be regulated by other factors independent of cell division. We have now identified a role for Tcf19 in maintaining ER homeostasis and preventing apoptosis. The basal expression of Tcf19 in islet may reflect its role in β-cell homeostasis rather than proliferation. However, we hypothesize that there is a secondary increase in Tcf19 during compensatory cell division, perhaps involving different signaling pathways and posttranslational modifications. Our study is limited by the correlative nature of expression changes in our in vivo and ex vivo models and the use of a proliferative cell line for mechanistic studies. Dynamic studies in mouse models and in isolated islet tissue will be needed to further clarify the role of Tcf19 in the native β-cell. The role of Tcf19 in other tissues also remains to be determined.

Tcf19 may play an important role in T1DM pathogenesis. Recently, a novel locus for type 1 diabetes was identified on human chromosome 6p in both the Wellcome Trust Case Control Consortium and the T1D Genetics Consortium populations (8). *TCF19* emerged as the most likely associated gene candidate. Three nonsynonymous single-nucleotide polymorphisms (SNPs) leading to amino acid changes in the FHA domain and the proline-rich domain of Tcf19 protein were in strong linkage disequilibrium with the linked SNPs. This genetic association implies an increased risk for T1DM in those carrying the mutation, but because T1DM is a multifactorial disease, mutations in *TCF19* alone are clearly not causal in isolation. A genetic predisposition to autoimmunity is already a clear factor in the development of T1DM, but only a small percentage of individuals with at-risk human leukocyte antigen alleles go on to develop disease (54). In addition, islet autoantibodies can be present in individuals who do not have T1DM or go on to develop it much later in life (54). Therefore, factors that mediate the β-cell response to lymphocyte infiltration and inflammation could increase the likelihood of progression to T1DM in at-risk individuals. Decreased Tcf19 function would lead to an increased susceptibility of the β-cell to apoptosis along with an inability to adequately compensate with increased β-cell proliferation. Combining this with a genetic predisposition to islet autoimmunity could result in more complete and rapid progression to a diabetic phenotype. Although it is certainly possible that Tcf19 plays an unknown role in the lymphocyte and in the autoimmune response in T1DM, our data support a model in which the association of *TCF19* with T1DM is at least partially due to its effects in the β-cell. Further investigation of the effects of the nonsynonymous SNPs on Tcf19 function will likely yield more insight into the validity of this mechanism of disease susceptibility.

Collectively, our data show that Tcf19 has a twofold functionality in the cell, as it is necessary for both proliferation and survival. These characteristics suggest that Tcf19 may positively impact β-cell mass in settings of increased insulin demand and β-cell stress. We propose that Tcf19 plays an important role in β-cell mass regulation and homeostasis in both T1DM and T2DM susceptibility.

## GRANTS

D. B. Davis acknowledges research support from the NIDDK (K08-DK-083442), the William and Judith Busse Women in Research Foundation, the University of Wisconsin School of Medicine and Public Health and Department of Medicine, Division of Endocrinology, the Wisconsin Alumni Research Foundation, and the Central Society for Clinical Research. D. B. Davis was also the recipient of a Pilot Award from the Integrated Islet Distribution Program that provided an allotment of human islets. M. E. Kimple has received research support from JDRF (17-2011-608). J. A. Lavine and K. A. Krautkramer have been supported by the Medical Scientist Training Program (T32GM08692). O. Cleaver and L. Marty-Santos have also been supported by the NIDDK [3R01-DK-079862-S1 (L. Marty-Santos), F31-DK-092098 (L. Marty-Santos), and 5-R01-DK-079862 (O. Cleaver)]. We also acknowledge statistical consultation through the Clinical and Translational Science Award program through National Center for Advancing Translational Sciences Grant UL1TR0000427.

## DISCLOSURES

The authors have nothing to disclose. The contents of this work do not represent the views of the Department of Veterans Affairs or the US Government.

## AUTHOR CONTRIBUTIONS

K.A.K., A.K.L., D.A.F., A.L.W., J.A.L., O.C., M.E.K., and D.B.D. contributed to the conception and design of the research; K.A.K., A.K.L., D.A.F., A.L.W., T.W.H., G.J.S., N.A.T., L.M.-S., and D.B.D. performed the experiments; K.A.K., A.K.L., D.A.F., A.L.W., T.W.H., G.J.S., N.A.T., L.M.-S., J.A.L., O.C., M.E.K., and D.B.D. analyzed the data; K.A.K., A.K.L., D.A.F., A.L.W., T.W.H., G.J.S., N.A.T., L.M.-S., J.A.L., O.C., M.E.K., and D.B.D. interpreted the results of the experiments; K.A.K., A.K.L., O.C., and D.B.D. prepared the figures; K.A.K., A.K.L., and D.B.D. drafted the manuscript; K.A.K., A.K.L., D.A.F., A.L.W., J.A.L., O.C., M.E.K., and D.B.D. edited and revised the manuscript; K.A.K., A.K.L., D.A.F., A.L.W., T.W.H., G.J.S., N.A.T., L.M.-S., J.A.L., O.C., M.E.K., and D.B.D. approved the final version of the manuscript.
